# Triglyceride−glucose index in the prediction of major adverse cardiovascular events in patients with type 2 diabetes mellitus after coronary artery bypass surgery: A retrospective cohort study

**DOI:** 10.3389/fendo.2022.1015747

**Published:** 2022-10-20

**Authors:** He Zhang, Hoshun Chong, Zeshi Li, Kai Li, Bomin Zhang, Yunxing Xue, Dongjin Wang

**Affiliations:** ^1^ Department of Cardiothoracic Surgery, Nanjing Drum Tower Hospital, Chinese Academy of Medical Sciences& Peking Union Medical College, Graduate School of Peking Union Medical College, Nanjing, China; ^2^ Department of Cardiothoracic Surgery, The Affiliated Drum Tower Hospital of Nanjing University Medical School, Nanjing, China

**Keywords:** triglyceride-glucose index, coronary artery bypass grafting, major adverse cardiovascular events, nomogram model, type 2 diabetes mellitus

## Abstract

**Background:**

Insulin resistance (IR) is a significant risk factor for cardiometabolic diseases and a defining feature of type 2 diabetes mellitus (T2DM). This study aimed to examine the potential value of triglyceride-glucose (TyG) index as a predictor of prognosis in coronary heart disease (CHD) patients with T2DM after coronary artery bypass grafting (CABG) surgery and to facilitate the identification of those at high risk of major adverse cardiovascular events (MACEs) for closer monitoring or possible early intervention.

**Methods:**

This study enrolled 386 T2DM patients who underwent CABG surgery at Nanjing Drum Tower Hospital. Patients were separated into two groups according to the median preoperative TyG Index. The Kaplan-Meier plot was used to compare the rate of MACEs-free survival in T2DM patients after CABG. The independent risk factors for the occurrence of MACEs were investigated using multivariate analysis. Nomogram was used to depict the predictive model.

**Results:**

Significantly more MACEs occurred in individuals with higher medians of the TyG index (65 (33.7%) vs. 39 (20.2%), p=0.003). TyG index [hazard ratio (HR) 12.926], LVEF [hazard ratio (HR) 0.916], and NYHA functional class III/IV [hazard ratio (HR) 4.331] were identified as independent predictors of MACEs incidence in post-CABG T2DM patients by multivariate analysis. The area under the curve (AUC) for predicting MACEs using the TyG index was 0.89 at five years. Combining the TyG index, LVEF, and NYHA functional class III/IV to build a novel risk assessment model for postoperative MACEs, the AUC climbed to 0.93 at five years. With AUCs, the nomogram comprised of the TyG index, LVEF, and NYHA functional class III/IV demonstrated strong specificity in the training and test sets.

**Conclusions:**

The incidence of MACEs is high among post-CABG T2DM patients with a high TyG index. TyG index improves the diagnostic accuracy of MACEs, especially at long-term follow-up. A high TyG index may serve as an early warning signal for individuals to undertake lifestyle adjustments that can reduce the progression or incidence of MACEs.

## Introduction

Coronary heart disease (CHD) continues to be the most significant worldwide cause of morbidity and mortality (1, 2). Patients with type 2 diabetes mellitus (T2DM) have a more significant risk of developing CHD (3–5). CHD death rates are twice higher among adults with DM than those without diagnosed DM, mainly due to an increased risk of stroke and myocardial infarction (MI) (6). Myocardial revascularization, including coronary artery bypass grafting (CABG) and percutaneous transluminal coronary intervention (PCI), is the primary therapeutic technique for CHD (7). However, despite the use of treatments currently indicated by guidelines, recurrent cardiovascular events (CVEs) in diabetic patients are higher than in non-diabetic patients (8).

Insulin resistance (IR) is a crucial risk factor for cardiometabolic illnesses and a defining characteristic of T2DM (9, 10). The triglyceride–glucose (TyG) index has become a reliable alternative diagnostic of IR with better performance than other IR markers such as homeostasis model assessment of IR (HOMA-IR) (11), glycosylated hemoglobin (HbA1c), triglyceride/high density lipoprotein (TG/HDL (12–15). In prior meta-analysis of high- and low-TyG patients without atherosclerotic cardiovascular

diseases (ASCVDs), a higher TyG index could be independently associated with a higher incidence of ASCVDs, CAD, and stroke in people without ASCVDs at baseline (16). TyG index is a reliable predictor of coronary artery disease prognosis.

CABG remains the treatment choice for diabetic individuals with severe coronary artery disease affecting multiple vessels over PCI (17). Even though several recent pieces of research have demonstrated the link between the TyG index and vascular disease, no studies have analyzed the prognosis of diabetic patients who underwent CABG.

This study aimed to examine the potential value of the TyG index as a predictor of prognosis in CHD patients with T2DM after CABG and to facilitate the identification of those at high risk of MACEs for closer monitoring or possible early intervention.

## Methods

### Patients

**Figure 1:**
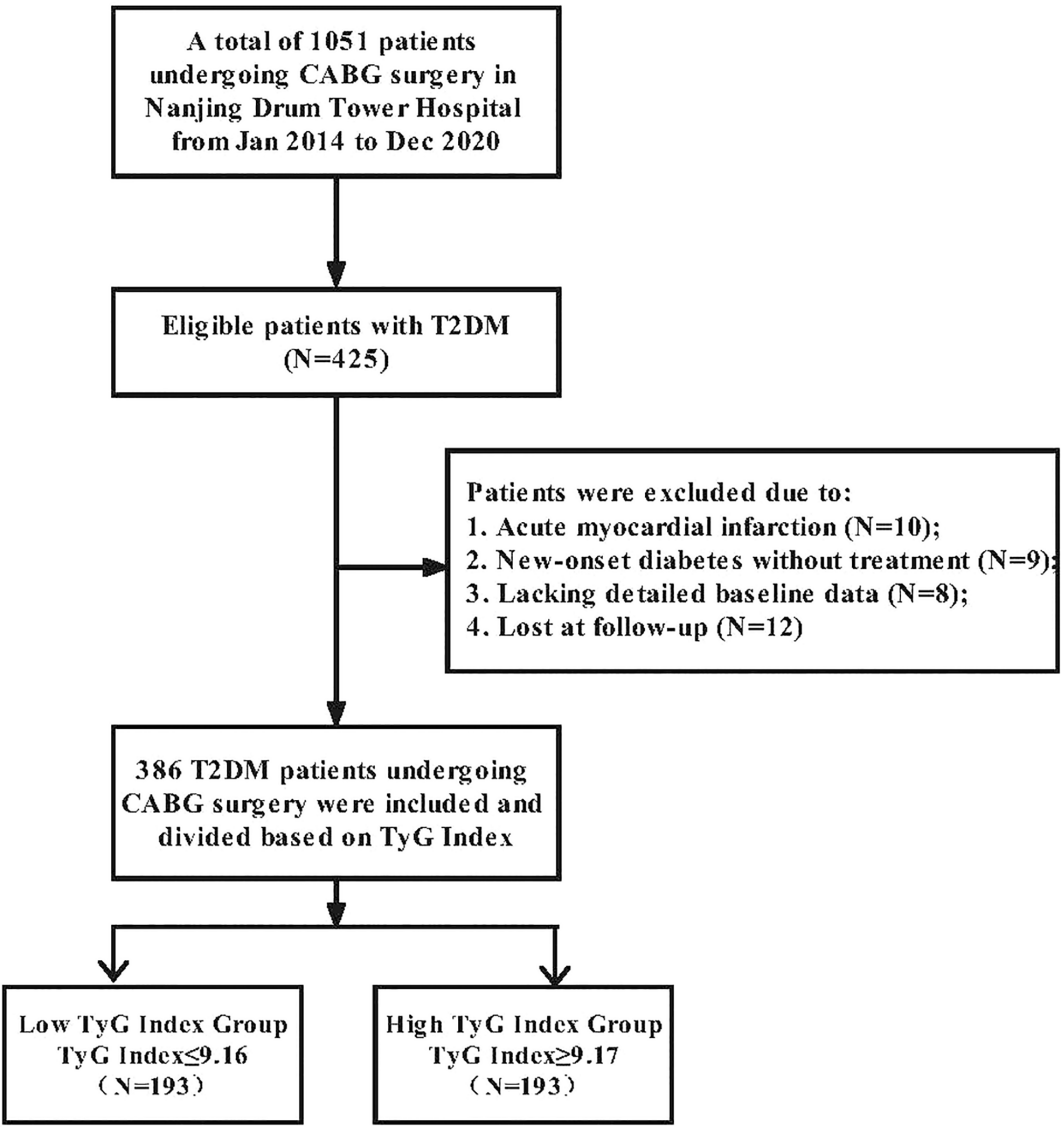
Flowchart of participant selection.

From January 2014 to December 2020, 1051 patients received isolated CABG surgery whether under cardiopulmonary bypass or off-pump at Nanjing Drum Tower Hospital. Four hundred twenty-five patients with T2DM were eligible for this study. The following factors determined exclusion: 1) Acute myocardial infarction; 2) New-onset diabetes untreated; 3) Absence of detailed baseline data; 4) Lost during follow-up. We separated 386 T2DM patients who underwent CABG surgery into two groups according to the median preoperative TyG Index. Medical assistants and nurses conducted telephone interviews monthly for patients’ follow-ups and documented the resulting data. The study flow chart is provided in **Figure 1**.

This study was approved by the Ethics Committee of Nanjing Drum Tower Hospital (NO.2020-281-01). Informed consent was exempt because of the retrospective nature of the study. The present study was conducted in accordance with the ethical principles stated in the Declaration of Helsinki (as revised in 2013).

### Data collection and definitions

All patient information was verified, rectified, and placed into a database at discharge time. Regular medical follow-up information was collected by phone and clinic visits.

T2DM was defined on the fasting blood-glucose (FBG) ≥7.0 mmol/L according to the American Diabetes Association’s standards of medical care (18) or self-reported physician diagnosis.

The TyG index was calculated as Ln [fasting triglycerides (mg/dL) × fasting glucose (mg/dL)/2] (19).

MACEs in this study included all-cause death, nonfatal myocardial infarction (MI), nonfatal stroke, revascularization (CABG or PCI) and rehospitalization for heart failure (HF).

The diagnosis of MI was according to the Fourth Universal Definition of Myocardial Infarction (20).

We used the New York Heart Association (NYHA) functional classification system to assess the severity of heart failure symptoms. Left ventricular function was expressed using ejection fraction of 2D echocardiography.

### Statistical analysis

Data were summarized using descriptive statistics, and numerical data were reported as means (SD). Using the Kolmogorov–Smirnov test, the distribution of continuous data variables was examined for normality. Using Fisher’s exact test, differences in the frequencies of categorical variables were also evaluated. All outcome indicators were compared across all patient groups. Fisher’s exact test was used to analyze variables that may have impacted the occurrence of MACEs in patients (univariate predictors). In order to identify independent predictors, the MACEs incidence predictors were subjected to multivariable logistic regression analysis. The final model includes the TyG index, NYHA functional class III/IV, and LVEF. In addition, the area under the curve (AUC) and ideal cut-off value were determined using receiver operating characteristic (ROC) curve analysis to evaluate the predictive efficacy of the TyG index, NYHA functional class III/IV, and LVEF for MACEs incidence. Survival was illustrated graphically using Kaplan–Meier curves. AUCs were utilized to determine the predictive value of the TyG index for MACEs.

The nomogram was constructed using the ‘rms’ R package and provides a risk score for each patient. The calibration curve was used to determine consistency between the predicted survival probability of the nomogram with bootstrap resamples. All statistical analyses were conducted with R (version 4.0.2) software, and a two-tailed P value of 0.05 was considered statistically significant.

## Results

### Baseline characteristics

The average age of the study participants was 66 years, and 276 (71.5%) were male. This study divided all patients into two groups based on the cut-off value of 9.17 for the TyG index. In two groups, the mean values of TyG index were 8.67 and 9.62, respectively. **Table 1** presents the study patients’ baseline clinical and laboratory characteristics according to the TyG index groups. Patients with a high TyG index were more likely to have had a stroke in the past. In proportion to the TyG index, BMI, TC level, LDL-C level, FPG level, and triglyceride level increased, whereas HDL-C level and LVEF declined. Furthermore, when the TyG index climbed, the proportion of three and above vascular disease and on-pump CABG also increased. Moreover, there was no significant change in the other variables, such as hospitalization duration and 30-day mortality.

**Table 1:** Baseline characteristics in T2DM patients with CABG surgery based on tertiles of TyG Index.

Variables	All patients (n=386)	Low TyG Index Group (n=193)	High TyG Index Group (n=193)	P-value
Male (n, %)	276 (71.5%)	138 (71.5%)	138 (71.5%)	1.000
Age (years)	66 (60-72)	66 (59-71.5)	66 (60-72)	0.806
BMI (kg/m^2^)	24.85 (22.77-26.82)	24.24 (22.49-26.00)	25.34 (23.13-27.34)	0.003
Past history				
Hypertension (n, %) Cerebral infarction (n, %)	288 (74.6%) 59 (15.3%)	146 (75.6%) 36 (18.7%)	142 (73.6%) 23 (11.9%)	0.640 0.066
Atrial fibrillation (n, %)	9 (2.3%)	5 (2.6%)	4 (2.1%)	0.736
Hyperthyroidism (n, %)	1 (0.3%)	0 (0.0%)	1 (0.5%)	0.317
PCI (n, %)	43 (11.1%)	20 (10.4%)	23 (11.9%)	0.627
LVEF, %	50 (49,58)	51 (48.5,59)	50 (50,57)	0.093
NYHA class				0.223
I-II	269 (69.7%)	129 (66.8%)	140 (72.5%)	
III-IV	117 (30.3%)	64 (33.2%)	53 (27.5%)	
Laboratory test				
Triglyceride	1.46 (1.10-1.93)	1.15 (0.95-1.46)	1.92 (1.46-2.56)	<0.001
Glucose	7.34 (6.08-10.10)	6.18 (5.02-7.22)	9.70 (7.50-12.90)	<0.001
TyG Index	9.17 (8.67-9.62)	8.67 (8.45-8.96)	9.62 (9.39-9.86)	<0.001
LDL-C	1.86 (1.42-2.41)	1.77 (1.30-2.18)	1.93 (1.49-2.42)	0.010
HDL-C	0.93 (0.64-1.07)	0.98 (0.82-1.09)	0.88 (0.73-1.02)	0.004
TC	3.42 (2.86-4.14)	3.28 (2.66-4.02)	3.60 (2.89-4.37)	<0.001
Creatinine	70.95 (58.53-89.00)	71.00 (58.50-88.00)	70.9 (58.35-89.80)	0.865
BUN	6.20 (5.00-7.70)	6.20 (4.90-7.60)	6.30 (5.05-7.80)	0.360
Off-pump CABG (n, %)	344 (89.1%)	176 (91.2%)	168 (87.0%)	0.191
Number of grafts				
I-II	32 (8.3%)	21 (10.9%)	11 (5.7%)	
III-IV	300 (77.7%)	146 (75.9%)	154 (79.8%)	
V-VI	54 (14.0%)	26 (13.5%)	28 (14.5%)	
In-hospital time (Day) Mortality (n, %)	21 (18-25) 18 (4.7%)	21 (17.5-25.5) 9 (4.7%)	21 (18-25) 9 (4.7%)	0.635 1.000

BMI, body mass index; PCI, percutaneous transluminal coronary intervention; LVEF, left ventricular ejection fraction; NYHA, New York Heart Association; LDL-C, low-density lipoprotein cholesterol; HDL-C, high-density lipoprotein cholesterol; TC, total cholesterol; BUN, blood urea nitrogen.

### Association between the TyG index and the risk of MACEs

**Figure 2:**
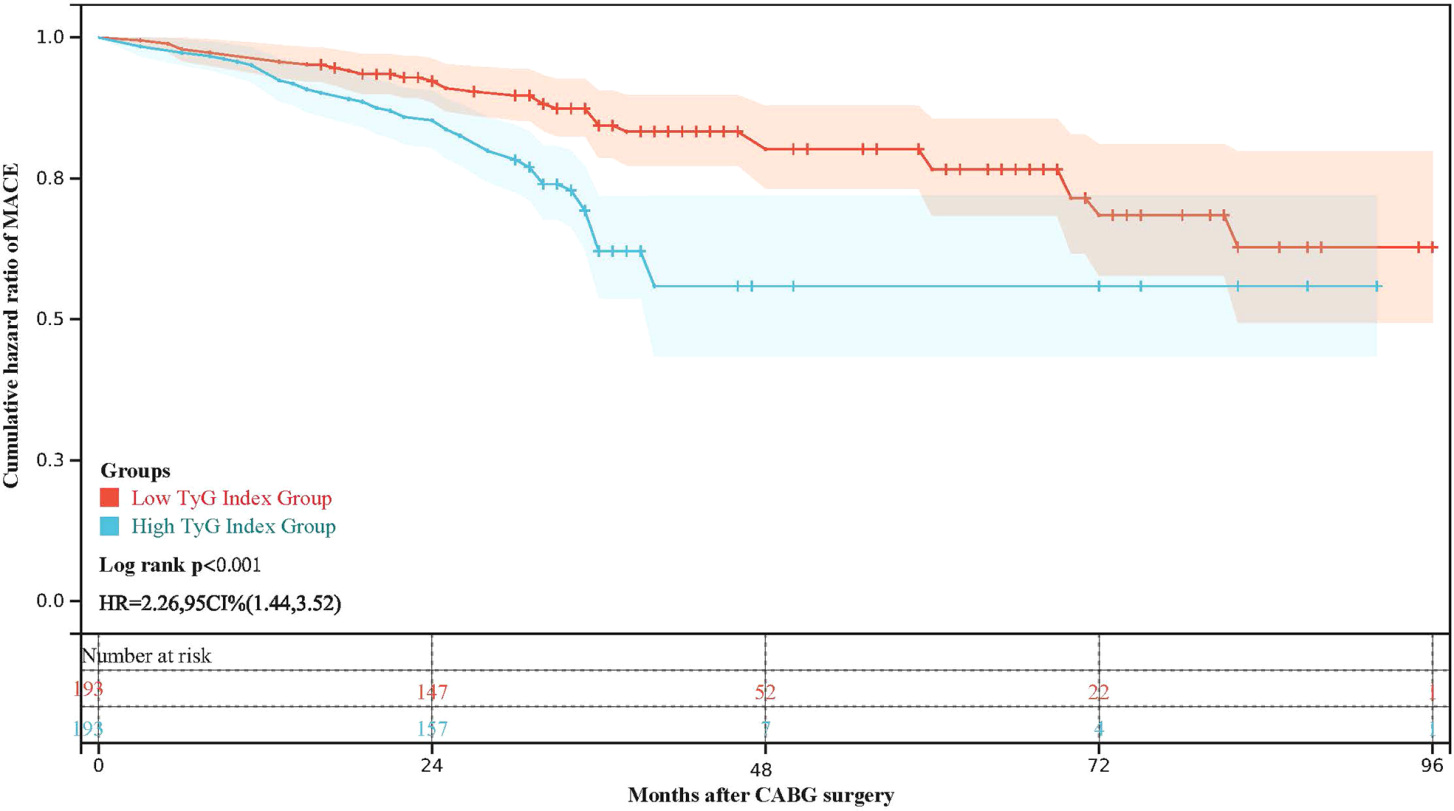
Kaplan-Meier survival curve for freedom from MACE in high TyG index group and low TyG index group.

During the follow-up period, 104 (26.9%) cases of MACEs in total. 38 (9.8%) cases of all-cause death occurred. 12 (3.1%) and 16 (4.1%) patients experienced nonfatal MI and stroke. 7 (1.8%) and 67 (17.4%) patients were re-admitted to the hospital for further revascularization and HF treatment. The incidence of MACEs was significantly higher in patients with higher medians of the TyG index (65(33.7%) vs. 39(20.2%), p=0.003). 13 (6.7%) of the deceased patients were in the low TyG Index Group, and 25 (13%) were in the high TyG index group. 46 (33.1%) patients with high TyG index were re-admitted to the hospital for HF treatment, while only 21 (10.9%) patients with low TyG index were re-admitted. All-cause death and rehospitalization for HF were statistically different between the two groups of patients. Although not statistically significant, high TyG index patients had a higher tendency to experience myocardial infarction (9(4.7%) vs. 3(1.6%), p=0.078). Other outcomes, including nonfatal stroke and the need for revascularization, showed no significant differences (**Table 2**). **Figure 2** depicts the Kaplan–Meier curves during the follow-up without a MACE survival curve according to the first occurrence of MACE for both TyG index groups. At five years, the incidence of MACE with a high TyG index was significantly higher (p<0.001) among T2DM patients.

**Table 2:** Clinical outcomes in T2DM patients with CABG surgery based on tertiles of TyG Index.

CV Outcomes	All patients(n=386)	Low TyG Index Group(n=193)	High TyG Index Group(n=193)	P-value
MACE	104 (26.9%)	39 (20.2%)	65 (33.7%)	0.003
All-cause death	38 (9.8%)	13 (6.7%)	25 (13.0%)	0.040
Nonfatal MI	12 (3.1%)	3 (1.6%)	9 (4.7%)	0.078
Nonfatal stroke	16 (8.3%)	6 (3.1%)	10 (5.2%)	0.307
Revascularization	7 (1.8%)	3 (1.6%)	4 (2.1%)	0.703
Rehospitalization for HF	67 (17.4%)	21 (10.9%)	46 (33.1%)	0.001

CV, cardiovascular; MI, myocardial infarction; HF, heart failure.

### TyG index, LVEF and NYHA functional class III/IV were predictors of MACEs occurrences

**Table 3** presents univariate and multivariate Cox proportional hazards regression analysis and predictors for composite MACEs. LVEF, NYHA functional class III/IV, TGs, FPG, TyG index, HDL-C, creatinine, BUN, off-pump CABG, ≥three-vessel disease were identified as risk factors for MACEs by univariate analysis (p<0.1). Further multivariate analysis of the risk factors mentioned above showed that TyG index (HR = 12.926, 95% CI = 3.457-48.323, P<0.001), LVEF (HR = 0.916, 95% CI = 0.886-0.946, P<0.001) and NYHA functional class III/IV (HR = 4.331, 95% CI = 2.410-7.781, P<0.001) were identified as independent predictors of MACEs occurrences in patients with T2DM underwent CABG.

**Table 3:** Univariate and multivariate analyses of MACE in T2DM patients with CABG surgery.

Variables	Univariate analysis			Multivariate analysis		
	HR value	95% CI	P-value	HR value	95% CI	P-value
Sexy (Male)	1.325	0.839-2.092	0.227			
Age	1.001	0.980-1.021	0.954			
BMI	1.015	0.960-1.074	0.599			
Hypertension	1.144	0.725-1.805	0.564			
Cerebral infarction	1.173	0.697-1.973	0.548			
Atrial fibrillation	3.607	1.579-8.240	0.002			
PCI	0.508	0.223-1.160	0.108			
LVEF	0.885	0.867-0.904	<0.001	0.916	0.886-0.946	<0.001
NYHA class (III-IV)	7.967	5.238-12.117	<0.001	4.331	2.410-7.781	<0.001
Triglyceride	1.435	1.210-1.703	<0.001	0.601	0.322-1.124	0.111
Glucose	1.118	1.077-1.160	<0.001	0.911	0.806-1.031	0.141
TyG Index	2.886	2.117-3.934	<0.001	12.926	3.457-48.323	<0.001
LDL-C	1.129	0.916-1.392	0.254			
HDL-C	0.370	0.150-0.911	0.030	0.756	0.236-2.423	0.638
	HR value	95% CI	P-value	HR value	95% CI	P-value
TC	1.118	0.948-1.317	0.184			
Creatinine	1.002	1.001-1.004	<0.001	1.001	0.998-1.003	0.583
BUN	1.078	1.033-1.125	0.001	0.990	0.916-1.069	0.798
Off-pump CABG	0.407	0.252-0.658	<0.001	0.717	0.420-1.222	0.221
I-II grafts	1			1		
III-IV grafts	3.099	0.978-9.814	0.054	0.770	0.234-2.527	0.666
V-VI grafts	4.563	1.361-15.299	0.014	0.979	0.280-3.420	0.974

BMI, body mass index; PCI, percutaneous transluminal coronary intervention; LVEF, left ventricular ejection fraction; NYHA, New York Heart Association; LDL-C, low-density lipoprotein cholesterol; HDL-C, high-density lipoprotein cholesterol; TC, total cholesterol; BUN, blood urea nitrogen.

**Figure 3:**
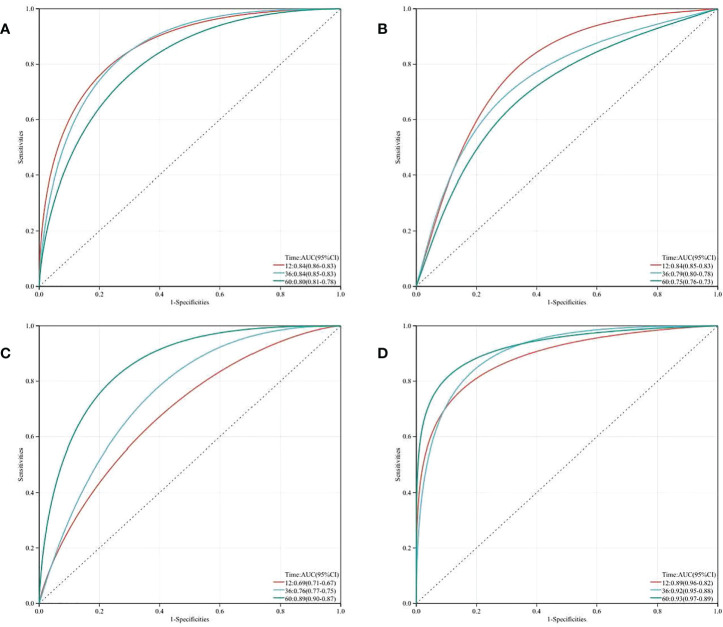
Time-dependent receiver operating characteristic curve (ROC) analysis with the area under the curve, sensitivity and specificity of LVEF **(A)**, NYHA class **(B)**, TyG Index **(C)** and LVEF + NYHA class + TyG Index **(D)** in predicting MACEs of T2DM patients after CABG surgery.

The predictive value of nomogram at 12/36/60 months postoperatively was evaluated by plotting time-dependent ROC curves for each of the three variables TyG Index, LVEF, and NYHA functional class III/IV. LVEF and NYHA functional class III/IV exhibited a better predictive value at 12 and 36 months postoperatively, whereas the TyG Index was more accurate at 60 months. The predictive value of the AUC of the TyG Index for the incidence of MACE was as high as 0.89. Then we combined TyG, LVEF, and NYHA functional class III/IV to build a novel risk assessment model for postoperative MACEs. At 12, 36, and 60 months, the AUC climbed to 0.89, 0.92, and 0.93, respectively. These findings indicate that the TyG index improves the diagnostic accuracy of this prediction model, especially at 60 months postoperatively (**Figure 3**).

**Figure 4:**
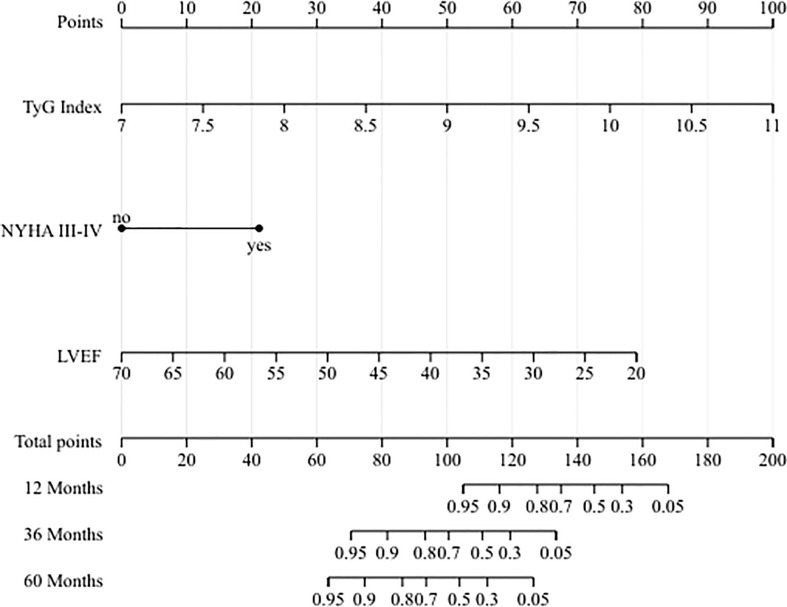
Nomogram for predicting 12-/36-/60-months incidence of MACEs.

**Figure 5:**
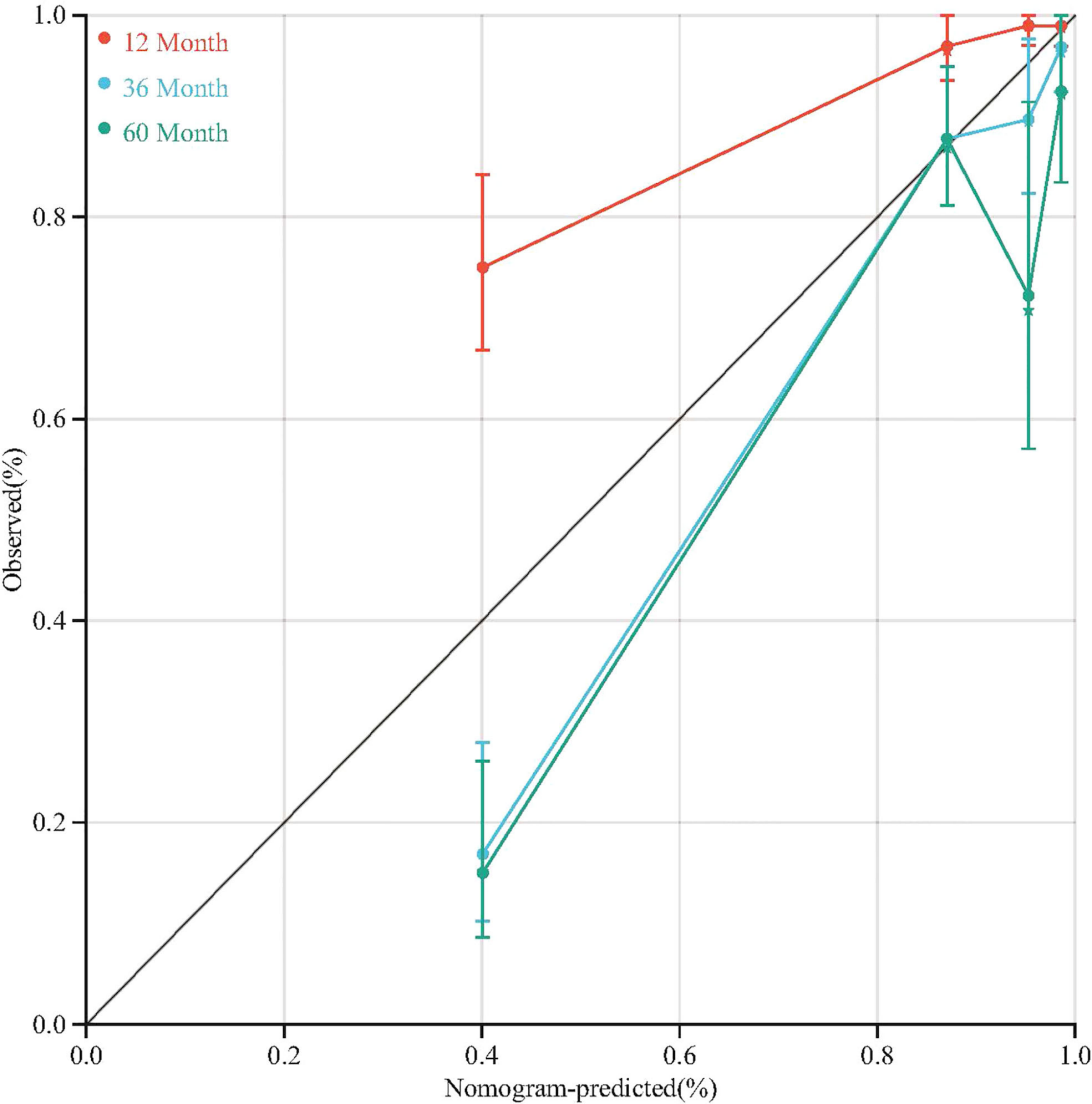
Calibration plots of the nomogram for 12-/36-/60-month incidence of MACEs.

To quantitative predict the incidence of MACEs in T2DM patients after CABG surgery, we established a prediction nomogram for MACEs was developed using the TyG index, NYHA functional class III/IV, and LVEF (**Figure 4**). All of these variables were assigned a score on the points scale. Summation over the variable points were summed, and a total point was obtained and located on the Total Points scale. A line was drawn straight down to the 12-/36-/60-months incidence of MACEs, and the estimated incidence at each time point is shown. Furthermore, the calibration plots showed good consistency between the nomogram predictions and actual observations for overall survival rate in each time point (**Figure 5**).

## Discussion

Our study assessed the predictive effectiveness of the TyG index for the occurrence of MACEs in patients with T2DM who had CABG. We found that diabetic CABG patients with a low TyG index had a higher long-term MACE-free rate than those with a high TyG index at a cut-off value of 9.17. Combining TyG, LVEF, and NYHA functional class III/IV, we constructed an effective model for evaluating the occurrence of MACEs.

Diabetes is an independent risk factor for coronary heart disease (9, 10, 21). More than 90% of diabetic patients are diagnosed with T2DM (22). Insulin resistance is a defining property of metabolic syndrome, a critical trait of T2DM, and a risk factor for cardiovascular events (23). Few pieces of research have examined insulin resistance as a cardiovascular risk factor. Srinivasan et al. examined 61 T2DM patients who underwent coronary arteriography and discovered that insulin resistance was positively associated with coronary risk severity (24). The San Antonio Heart Study demonstrated a substantial relationship between insulin resistance and the risk of cardiovascular disease (25). In a large observational trial, Hedblad B et al. found that insulin-resistant patients’ relative risk for cardiovascular events and death was twofold higher (26). Recent studies have demonstrated the close link between the TyG index and the homeostasis model for measuring insulin resistance (HOMA-IR) (27, 28). In addition, the predictive value of the TyG index for IR was superior to that of the HOMA-IR (11). Indicating TyG index, a hematological indicator of IR that is straightforward, practical, and stable, is a reliable predictor of coronary artery disease prognosis. Others have shown that a higher TyG index is associated with an increased risk of significant adverse cardiac and cerebrovascular events in patients having a percutaneous coronary intervention for ST-elevation myocardial infarction (STEMI) (29). Although multiple recent studies have proven the association between the TyG index and vascular disease (30, 31), no studies have examined the prognosis of diabetes patients with CABG. Therefore, we studied its role further in CABG patients with T2DM and discovered that it had potential prognostic value.

The effect of diabetes on post-CABG short-term mortality was minimal. Both diabetic and non-diabetic individuals have comparable in-hospital and 1-year mortality rates (32). Indeed, LVEF and NYHA functional classes significantly impact in-hospital and 1-year mortality rates in post-CABG patients. Following these findings, we found that LVEF and NYHA functional class III/IV did well predicting the occurrence of MACEs at 12 and 36 months. Our results demonstrated that multivariate Cox regression analysis identified LVEF and NYHA functional class III/IV as independent risk factors for the development of MACEs. In addition, throughout early and middle-term follow-up, LVEF and NYHA categorization had a higher diagnostic value for the occurrence of MACEs, with AUCs of 0.84 for both. Despite no significant difference between high and low TyG in-hospital mortality, we found statistical significance in the absence of MACEs based on variations in the TyG index. The most substantial impact of diabetes mellitus is definitely on long-term outcomes. Also, LVEF and NYHA functional class III/IV significantly impact early- and mid-term outcomes (33, 34). A unique risk assessment approach for postoperative MACEs compatible with early-, mid-, and long-term follow-up is required. Therefore, we combined the TyG index, LVEF, and NYHA functional class III/IV, three independent risk variables, to find a novel assessment tool for predicting the risk of MACEs in post-CABG diabetic patients.

In addition, no clinical scores can predict the long-term risk of MACEs in T2DM patients after CABG surgery. The nomogram constructed from numerous independent predictors has been recognized as a practical and effective illness prediction tool. As a result, we developed a nomogram based on the multivariate analysis to predict MACEs at 12,36 and 60 months, which showed good accuracy and consistency. However, due to the limited number of cases and single-center investigation, this nomogram has not been externally validated, and the accuracy of its predictions in diverse populations requires further study. Further research is required to evaluate if diabetic persons with a high TyG index should be included in risk stratification algorithms for the occurrence of MACEs in post-CABG patients. In future clinical practice, a high TyG index could serve as an early warning signal for individuals to initiate lifestyle modifications that can minimize the progression or incidence of MACEs.

## Conclusions

In conclusion, the morbidity of MACEs is substantial in T2DM patients with a high TyG index having CABG. TyG index, LVEF, and NYHA functional class III/IV were identified as independent risk variables for the occurrence of MACEs in T2DM patients after CABG, according to multivariate analysis. In addition, a unique nomogram for predicting the incidence of MACEs comprised of the three independent risk variables was demonstrated to be a possible indicator for early intervention.

## Data availability statement

The raw data supporting the conclusions of this article will be made available by the authors, without undue reservation.

## Ethics statement

This study was approved by the Ethics Committee of Nanjing Drum Tower Hospital (NO.2020-281-01). Written informed consent for participation was not required for this study in accordance with the national legislation and the institutional requirements.

## Author contributions

Conception and design: HZ and DW. Administrative support: YX and DW. Provision of study materials or patients: BZ and DW. Collection and assembly of data: HZ and ZL. Data analysis and interpretation: HZ, HC, ZL, and KL. Manuscript writing: all authors. Final approval of manuscript: all authors.

## Funding

This work was supported by the National Natural Science Foundation of China (Nos.81970401, 82100508), Jiangsu Provincial Key Medical Discipline (ZDXKA2016019), and Natural Science Foundation of Jiangsu Province (BK20210014).

## Conflict of interest

The authors declare that the research was conducted in the absence of any commercial or financial relationships that could be construed as a potential conflict of interest.

## Publisher’s note

All claims expressed in this article are solely those of the authors and do not necessarily represent those of their affiliated organizations, or those of the publisher, the editors and the reviewers. Any product that may be evaluated in this article, or claim that may be made by its manufacturer, is not guaranteed or endorsed by the publisher.
